# Supplementary material for: Disease severity dictates SARS-CoV-2-specific neutralizing antibody responses in COVID-19
Source: Signal Transduct Target Ther. 2020 Sep 2;5:180. doi: 10.1038/s41392-020-00301-9 (PMC7464057; doi:10.1038/s41392-020-00301-9)
Supplement: Supplementary file 1 — Supplementary materials [file 41392_2020_301_MOESM1_ESM.docx]

Supplementary Materials for

Disease severity dictates SARS-CoV-2-specific neutralizing antibody responses in COVID-19

Xiangyu Chen, Zhiwei Pan, Shuai Yue, Fei Yu, Junsong Zhang, Yang Yang, Ren Li, Bingfeng Liu, Xiaofan Yang, Leiqiong Gao, Zhirong Li, Yao Lin, Qizhao Huang, Lifan Xu, Jianfang Tang, Li Hu, Jing Zhao, Pinghuang Liu, Guozhong Zhang, Yaokai Chen, Kai Deng & Lilin Ye

Correspondence to: yelilinlcmv@tmmu.edu.cn

**This PDF file includes:**

Materials and Methods

Figures. S1 to S2

Tables S1

**Materials and Methods**

**Human samples.** The 59 COVID-19 recovered patients enrolled in the study were provided written informed consent and from different sources. The sera of the severe, moderate and mild patients were obtained from Guangzhou Eighth People’s Hospital. The sera of the asymptomatic patients were obtained from Chongqing Public Health Medical Center. Healthy control subjects were 4 adult participants in the study. The study received IRB approvals at Guangzhou Eighth People’s Hospital (KE202001134) and Chongqing Public Health Medical Center (2020-023-01-KY).

**ELISA.** As previously described^15^, 50 ng of SARS-CoV-2 S1 protein (Sino Biological, 40591-V08H) or SARS-CoV-2 RBD protein (Sino Biological, 40592-V08B) or SARS-CoV-2 S2 protein (Sino Biological, 40590-V08B) in 100 μl PBS per well was coated on ELISA plates (Costar, 42592) overnight at 4^o^C. The ELISA plates were blocked for 1 hour with 100 μl blocking buffer (5% FBS and 0.1% Tween 20 in PBS) and then incubated with diluted patient or healthy control sera in 100 μl blocking buffer for 1 hour. After washing with PBST buffer (0.1% Tween 20 in PBS), the ELISA plates were incubated with anti-human IgG HRP antibody (Bioss Biotech, 0297D) for 45 min, followed by PBST washing and addition of TMB buffer (Beyotime, P0209). The ELISA plates were allowed to react for 5-10 min and stopped by 1 M HCl stop buffer. The optical density (OD) value was detected at 450 nm.

**ELISA-based RBD-ACE2 binding inhibition assay.** As previously described^15^, 200 ng of ACE2 protein (Sino Biological, 10108-H08H) in 100 μl PBS per well was coated on ELISA plates overnight at 4^o^C. The ELISA plates were blocked for 1 hour with 100 μl blocking buffer (5% FBS and 0.1% Tween 20 in PBS); meanwhile, 50 μl 10-fold diluted patient or healthy control sera were incubated with 7.5 ng SARS-CoV-2 RBD-mouse FC protein (Sino Biological, 40592-V05H) in 50 μl blocking buffer for 1 hour. Then, the incubated sera/SARS-CoV-2 RBD-mouse FC protein mixture was added into the ELISA plates and allowed to develop for 30 min, followed by PBST washing and incubation with anti-mouse FC HRP antibody (Thermo Fisher Scientific, A16084) for 30 min. Next, the ELISA plates were washed with PBST and treated with TMB buffer (Beyotime, P0209). After 5 min, the ELISA reaction was stopped by 1 M HCl stop buffer and determined at 450 nm. The RBD-ACE2 binding inhibition score was calculated as: 100 × (1 - (OD450 value of patient sera / OD450 value of healthy control sera)).

**Pseudovirus neutralization assay.** The pseudovirus neutralization assay was previously described^15,32^. Briefly, HEK-293T cells were transfected with pLenti-luciferase, psPAX2, and 2019-nCov S plasmids by using *Trans*IT-293 Transfection reagent (Mirus, MIR 2700). After 12 hours, the culture media was changed to fresh media. And at 64 hours after transfection, the culture supernatants containing SARS-CoV-2 typed pseudovirus were harvested. Next, 200-fold diluted patient or healthy control sera were mixed with SARS-CoV-2 typed pseudovirus for 1 hour at 37^o^C. Then, the ACE2-expressing HEK-293T (ACE2/293T) cells were incubated with the sera/pseudovirus mixture overnight and then cultured with fresh media. At 40 hours after the mixture incubation, the luciferase activity of SARS-CoV-2 typed pseudovirus-infected ACE2/293T cells were measured by a luciferase reporter assay kit (Promega, E1910).

**SARS-CoV-2 serum neutralization assay.** Patient sera were diluted in DMEM (40 fold-dilution) and mixed with an equal volume of 80-100 PFU SARS-CoV-2 (EPI_ISL_444969) for 1 h at 37°C. Serum-virus mixture were then added to the Vero E6 cell monolayers in 48-well plates and incubated at 37°C in 5% CO_2_ for 1 h. After removing the inocula, plates were overlaid with culture medium and cultured at 37°C for 48 h. Subsequently, viral RNA from the cultural supernatants was extracted and the viral RNA copies were determined by quantitative PCR according to the viral detection kit’s protocol (DAAN Gene Co., Ltd. of Sun Yat-Sen University). All experiments related to authentic viruses were performed in the certified BSL-3 facility of Sun Yat-sen University. The SARS-CoV-2 viral RNA fold reduction = 2^(CT value of sample - CT value of mock)^.

**Depletion of SARS-CoV-2 S protein-specific antibodies.** Firstly, SARS-CoV-2 S1 protein (Sino Biological, 40591-V08H) or SARS-CoV-2 RBD protein (Sino Biological, 40592-V08B) or SARS-CoV-2 S2 protein was conjugated with biotin by following the manufacture’s protocol (Thermo Fisher Scientific, A39257). Then, biotin-conjugated proteins were incubated with BeaverBeads Mag Streptavidin Matrix (Beaver, 22305) at 4^o^C for 1.5 hours. After washing with PBS, the SARS-CoV-2 S protein coupled beads were next incubated with diluted patient sera at 4^o^C for 1.5 hours. Then, the supernatants were harvested and quality controlled by ELISA assays for SARS-CoV-2 S proteins.

**Statistics.** The SARS-CoV-2 antibody titers or virus neutralizing function of the sera belonging to patients with different severity were compared with the one-way ANOVA test (Tukey’s multiple comparisons test). The cutoff value in each pseudovirus neutralizing function assay was determined by the ROC curve analysis and was of the highest likelihood ratio. Correlations between different SARS-CoV-2 antibody titers or between SARS-CoV-2 antibody titers and pseudovirus titers or between SARS-CoV-2 antibody titers and SARS-CoV-2 virus titers were analyzed using Pearson’s correlation coefficient. *P* values less than 0.05 were defied as statistically significant. Prism 6 software was used for statistical analysis.

**
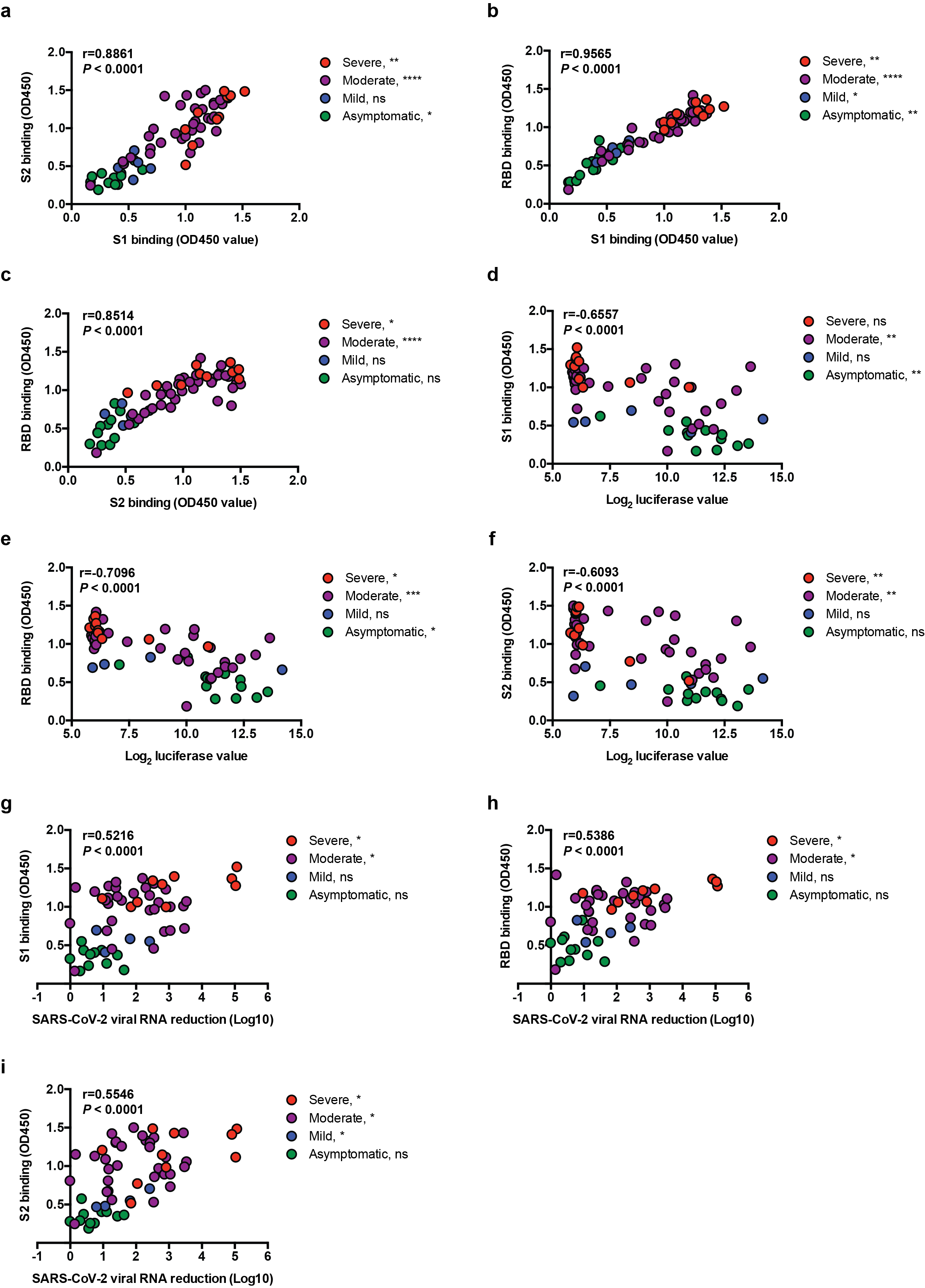
**

**Figure. S1. Correlation analyses of SARS-CoV-2 antibody titers and neutralizing functions in COVID-19 recovered patients.**

**a-c**, Correlations between SARS-CoV-2 S1 antibody titer and SARS-CoV-2 S2 antibody titer (a), SARS-CoV-2 S1 antibody titer and SARS-CoV-2 RBD antibody titer (b) and SARS-CoV-2 S2 antibody titer and SARS-CoV-2 RBD antibody titer (c) in the 59 COVID-19 recovered patients. **d-f**, Correlations between SARS-CoV-2 S1 antibody titer and SARS-CoV-2 pseudovirus neutralizing function (d), SARS-CoV-2 RBD antibody titer and SARS-CoV-2 pseudovirus neutralizing function (e) and SARS-CoV-2 S2 antibody titer and SARS-CoV-2 pseudovirus neutralizing function (f) in the 59 COVID-19 recovered patients. **g-i**, Correlations between SARS-CoV-2 S1 antibody titer and SARS-CoV-2 virus neutralizing function (g), SARS-CoV-2 RBD antibody titer and SARS-CoV-2 virus neutralizing function (h) and SARS-CoV-2 S2 antibody titer and SARS-CoV-2 virus neutralizing function (i) in the 59 COVID-19 recovered patients. The *P* value and r value at the top left of each panel assess the correlation between the indicated two parameters in the total 59 patients. The asterisks adjacent to severity at the top right of each panel assess the correlation between the indicated two parameters in patients of indicated severity. **P* < 0.05, ***P* < 0.01, ****P* < 0.001 and *****P* < 0.0001. Not significant, ns.

**
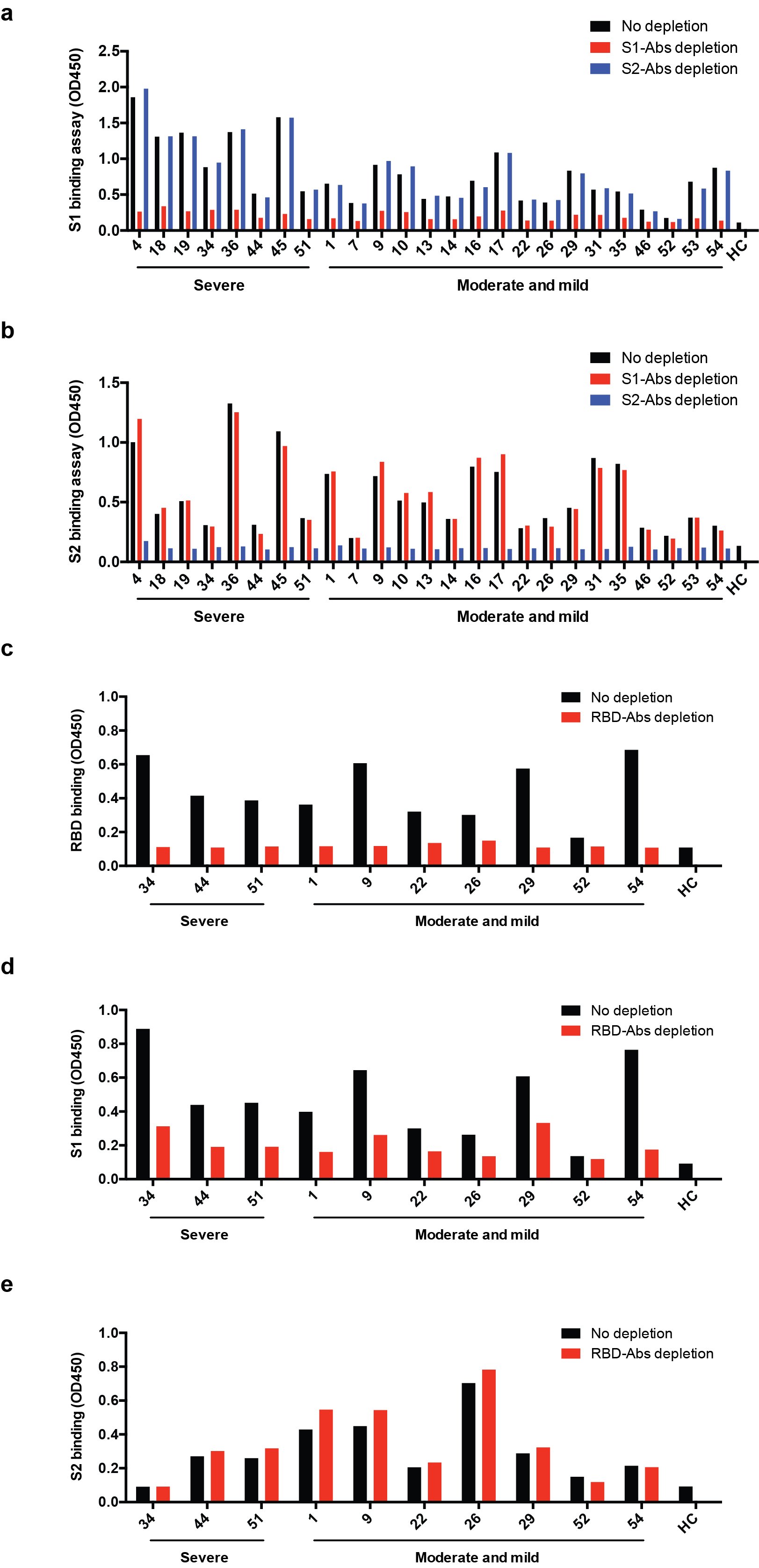
**

**Figure. S2. Depletion efficiencies of SARS-CoV-2 S1, S2 and RBD antibodies in patient sera.**

**a, b**, ELISA binding assays of 1500-fold diluted COVID-19 patient sera (no depletion) or S1 antibodies-depleted sera (S1-Abs depletion) or S2 antibodies-depleted sera (S2-Abs depletion) to ELISA plates coating of SARS-CoV-2 S1 (a) and S2 (b) proteins. HC, healthy control. **c-e**, ELISA binding assays of 1500-fold diluted COVID-19 patient sera (no depletion) or RBD antibodies-depleted sera (RBD-Abs depletion) to ELISA plates coating of SARS-CoV-2 RBD (c), S1 (d) and S2 (e) proteins. HC, healthy control.


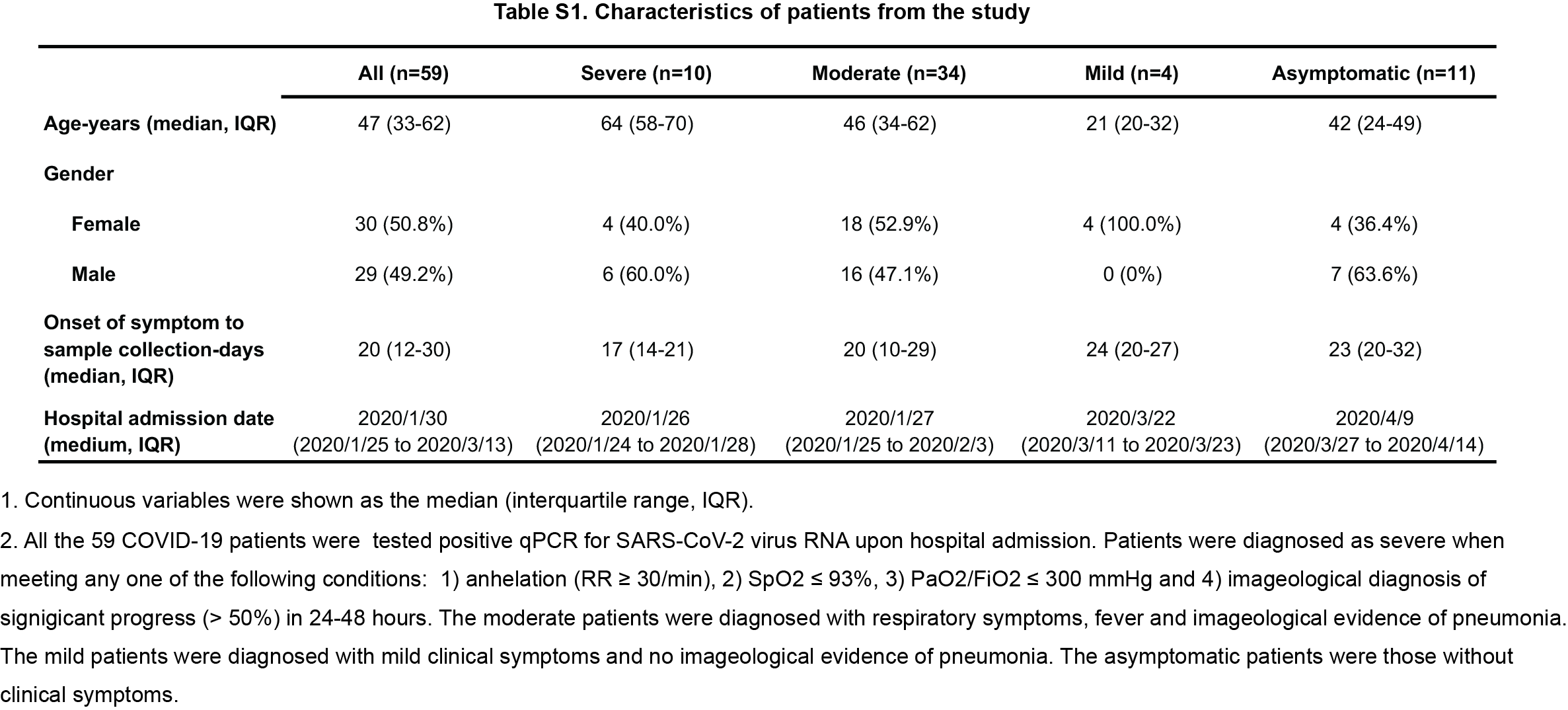


**Table S1. Characteristics of patients from the study.**
